# Carcass Characteristics and Meat Quality of Two Serbian Indigenous Chicken Breeds: Comparative Assessment of Banat Naked Neck and Svrljig Hen

**DOI:** 10.3390/foods15091546

**Published:** 2026-04-29

**Authors:** Zdenka Škrbić, Veselin Petričević, Simeon Rakonjac, Vladimir Dosković, Maja Petričević, Nataša Tolimir, Miloš Lukić

**Affiliations:** 1 Institute for Animal Husbandry, Autoput za Zagreb 16, 11080 Belgrade, Serbia; veselin5@live.com (V.P.); majanovakovic@live.com (M.P.); miloslukic.izs@gmail.com (M.L.); 2 Faculty of Agronomy, University of Kragujevac, Cara Dušana 34, 32102 Čačak, Serbia; simeonr@kg.ac.rs (S.R.); vladosko@kg.ac.rs (V.D.); 3 Institute for Science Application in Agriculture, Bul. Despota Stefana 68b, 11000 Belgrade, Serbia; tolimirnatasa68@gmail.com

**Keywords:** meat quality, indigenous chicken breed, Banat Naked Neck, Svrljig hen, slaughter age

## Abstract

Local chicken breeds are increasingly being reconsidered as a means to produce distinctive meat in non-conventional systems while also supporting the conservation of endangered genetic resources. This study compared Banat Naked Neck (BNN) and Svrljig hen (SH), two Serbian indigenous breeds, reared under identical pasture-based conditions and slaughtered at 12 or 14 weeks. Carcass traits, including linear measurements and carcass composition, were evaluated in 40 males (10 per breed per age), while breast and thigh-with-drumstick meat quality (proximate composition and fatty acid profile) was analysed in 80 samples (10 per tissue per group). Data were analysed using two-way ANOVA, and multivariate patterns were explored using PCA and residual Spearman correlation analysis. BNN and SH showed similar slaughter weights, whereas slaughter at 14 weeks increased carcass conformation measures and conformation indices (*p* < 0.05). Breed differences were most evident in carcass part distribution and tissue partitioning within cuts: BNN had a higher breast proportion and breast meat yield, whereas SH meat was leaner and thigh with drumstick meat showed higher Σn − 3 and a more favourable Σn − 6/Σn − 3 ratio. PCA indicated clearer breed separation in thigh meat than in breast meat, consistent with the univariate lipid results, and residual correlations highlighted expected allocation trade-offs among carcass and cut components. Overall, slaughter at 14 weeks improved carcass value, and both breeds offer complementary traits for market-oriented conservation through use.

## 1. Introduction

Chicken meat is an important source of animal protein for the growing human population. It provides high-quality nutrients and generally has a lower fat content compared with meat from other livestock species [1]. Its convenience for consumers of all ages and health statuses, along with the absence of major religious restrictions, has contributed to its global popularity. In recent decades, genetic progress in meat-type chickens has mainly focused on rapid growth and increased breast yield to meet market demand for cut-up portions, particularly breast meat, which is the most economically valuable part of the carcass [2]. As a result, less productive pure breeds, including indigenous and local breeds, have been largely neglected, threatening their long-term survival.

Alongside efficiency gains, intensive selection for rapid growth has been associated with quality and welfare challenges. Several studies report that the occurrence of meat abnormalities such as woody breast and pale, soft, exudative (PSE) traits is related to genotype and growth rate [3,4]. These defects may negatively affect the nutritional value of meat [5] and impair technological properties relevant to processing, resulting in economic losses for the poultry industry [6]. Moreover, selection for rapid growth has been linked to reduced adaptability under suboptimal rearing conditions [7] and a higher incidence of metabolic disorders [8,9]. Meanwhile, consumers increasingly demand products aligned with healthy diets, improved animal welfare, and transparent origin and safety. Conventional broiler meat remains central to human nutrition, but these expectations have stimulated interest in diversified poultry markets and alternative systems that produce meat with specific quality attributes.

Locally adapted and indigenous chicken breeds may play an important role in developing such non-conventional production systems. These breeds typically require relatively low inputs in housing, nutrition, and care, which limits productivity but may yield meat with characteristics associated with traditional production [10]. Meat quality in alternative systems is influenced by breed, rearing conditions (including opportunities for activity and natural feeding), and slaughter age [10,11]. Therefore, breed-specific and age-dependent data are essential for designing viable production strategies for indigenous genetic resources.

The Banat Naked Neck (BNN) and Svrljig hen (SH) are indigenous Serbian chicken breeds, and their conservation is of national importance. Due to their small population sizes, both breeds are considered endangered [12]. The estimated population of BNN (approximately 1000–2000 birds) is larger than that of SH (approximately 300–500 birds), and BNN has expanded beyond Vojvodina into other regions of Serbia, whereas SH remains mainly concentrated in eastern Serbia, its region of origin. Both breeds are medium-sized and dual-purpose, typically reaching body weights of about 2–3 kg (BNN) and 1.5–2 kg (SH) [13]. Conservation of these genetic resources is therefore imperative, and the development of niche poultry products, such as meat of specific quality from indigenous breeds, represents a potential pathway to support sustainable use. Market-oriented non-conventional production based on these breeds could also provide socioeconomic benefits for rural households by creating additional income opportunities and supporting local development [14].

Despite their importance for biodiversity and rural livelihoods, BNN and SH have been examined in relatively few studies, consistent with their limited populations. Available reports on BNN indicate that carcass yield and conformation do not differ substantially among colour varieties (white, black, grey) [15], and that compared with a moderate-growth hybrid (Redbro), BNN exhibits poorer carcass conformation and lower yields [16]. The same study suggests that slaughter at an older age may increase thigh circumference and carcass yield in BNN chickens. Regarding lipid composition, free-range BNN chickens slaughtered at 84 days reportedly showed a less favourable fatty acid profile (lower PUFA and Ʃn − 3 fatty acids) compared with conventionally reared hybrids slaughtered at 42 days [17]. Only one study has directly compared BNN and SH for carcass traits, reporting significant differences at 84 days in the proportions of drumsticks and thighs and of back and pelvis between breeds [18]. However, to the best of our knowledge, no study has comprehensively compared the meat quality of these two indigenous breeds. Our study is also novel in its practical conservation context: in endangered populations, the best females are primarily retained for reproduction to support breed preservation, while males are the category with the greatest immediate relevance for potential meat production. Therefore, focusing on males is not only biologically justified but also highly relevant for evaluating whether these local genetic resources may have added value in the production of meat of specific quality, which could further support their sustainable preservation and promotion.

To support the conservation and sustainable use of BNN and SH in non-conventional production systems, more robust and comprehensive datasets are required. Importantly, these breeds are not subject to intensive selection for genetic improvement; consequently, their reproductive and production performance may vary considerably by locality and rearing conditions [19], which can contribute to inconsistent findings across studies. Therefore, the objective of this study was to address existing knowledge gaps by comparing BNN and SH chickens in terms of carcass conformation measurements, proportions of carcass parts, breast and thigh with drumstick tissue, proximate and intramuscular fatty acid composition. Comparisons were made at two slaughter ages: 12 weeks, commonly used in previous studies, and 14 weeks, to assess whether a slightly older slaughter age improves carcass conformation and yield and to determine its effects on meat quality. By characterising these breeds under defined growing conditions, this study aims to provide evidence supporting their sustainable use and economic valorisation, thereby contributing to their long-term conservation.

## 2. Materials and Methods

### 2.1. Design, Birds and Management

The experiment and experimental procedures were evaluated by the Ethics Committee of the Institute for Animal Husbandry (Belgrade, Serbia) and approved by the Veterinary Directorate in MAFW of the Republic of Serbia (Approval No: 002097079/2025).

A total of 240 chickens of both sexes were used (120 Banat Naked Neck, BNN; 120 Svrljig hen, SH). The birds originated from eggs obtained from parent flocks maintained at the Institute for Animal Husbandry (Belgrade, Serbia) and were incubated under standard conditions. Day-old chicks were placed in group pens with chopped straw litter, with 3 replicates per breed (40 chicks per replicate). The chicks were hatched in spring and reared indoors for the first 3 weeks under appropriate ambient conditions (primarily temperature) and lighting (23L:1D during the first three day; 16L:8D from days 4 to 21). Subsequently, the birds were transferred to mobile group cages placed on pasture (3 replicates per breed). Stocking density was 8 birds/m^2^. The birds were exposed to ambient outdoor temperature and natural daylight. The cages were equipped with round drinkers, feeders, and perches, and provided protection from sun and adverse weather. Regular movement of the cages ensured continuous access to fresh pasture.

The basal diet consisted of a complete corn–soybean meal-based mash formulated to contain 18.3% crude protein and 12.2 MJ/kg metabolisable energy, and was offered ad libitum, supplemented with forage available from the pasture. The pasture utilised in this trial is as a lowland semi-natural grassland with moderate biomass productivity. The botanical composition is dominated by the *Poaceae* family, primarily represented by *Festuca pratensis* and *Lolium perenne*. The *Fabaceae* component is subordinate, with *Trifolium pratense* as the dominant legume, while the ruderal fraction consists primarily of *Asteraceae* species, most notably *Bellis perennis* and *Taraxacum officinale*. All birds received the same diet and occupied the same pasture during the entire experimental period.

### 2.2. Data Collection

#### 2.2.1. Slaughter Procedure

After the birds reached the target slaughter ages (12 and 14 weeks), the selected birds were slaughtered according to standard procedures. The 12-week slaughter age was chosen to allow comparison with previous studies on these breeds, while 14 weeks was included to assess whether a slightly extended rearing period improves carcass conformation and yields, and affects meat quality traits. At each slaughter age, a random sample of 10 male chickens per breed (n = 40 males total; 2 breeds × 2 ages × 10 birds) was selected for carcass conformation, carcass yield and composition, and meat quality analyses. By selecting only male chickens in our study we additionally homogenised the sample by eliminating the effect of gender, which is significant for yield and meat quality. An additional reason, in dual-purpose breeds, males that are not selected for reproduction can be used for meat production. Feed was withdrawn 8 h prior to slaughter. Birds were slaughtered and processed manually, after which carcasses were chilled at 4 °C.

#### 2.2.2. Carcass Conformation Measurements

Carcass conformation was evaluated on processed, chilled carcasses using morphometric measurements according to Pavlovski et al. [20]: shank length (SL; distance between the foot pad and hock joint on the metatarsus of the right leg, measured with a calliper), keel length (KL; distance between cranial and caudal ends of the keel bone), breast depth (BD; calliper placed between cranial keel and dorsal surface above the first thoracic vertebrae), breast angle (BA; measured with a protractor at 1–1.5 cm from the cranial part of the sternum), and thigh girth (TG; measured by tape at the widest part of the right thigh). Conformation indices were expressed as the ratio of slaughter weight (SW) to each measure (e.g., SW/SL, SW/KL, SW/BD, SW/TG; g/mm).

#### 2.2.3. Carcass Processing, Cut-Up Parts, and Dissection Yields

Carcasses were cut into main parts in accordance with Commission Regulation (EC) [21], and weights were recorded. Conventionally dressed carcass (CC) weight was recorded as: carcass with head, neck, lower legs, and edible internal organs. Relative proportions of carcass parts (head, neck, back, pelvis, wings, breast, and thigh with drumstick) and edible internal organs (heart, liver, and gizzard) were calculated relative to SW. Breast and thigh with drumstick were dissected into muscle, skin, and bones, and component weights were recorded. Component yields were expressed relative to the cut and conventionally dressed carcass (CC) weights.

#### 2.2.4. Proximate Composition and Fatty Acid Profile

Proximate and fatty acid composition were determined in breast (white meat) and thigh with drumstick (dark meat). A total of 80 samples were analysed (10 breast samples and 10 thigh with drumstick samples per breed × age group). For both proximate and fatty acid analyses, samples were taken from homogenised muscle tissue of the entire breast and the entire thigh-with-drumstick cut, rather than from a specific anatomical subregion.

The proximate chemical composition of meat was determined in the following manner: dry-matter content was determined by drying samples at 103 ± 2 °C [22]; crude protein content was determined by the Kjeldahl method, i.e., quantified the total nitrogen content in the sample and multiplied with a factor of 6.25 to estimate the crude protein content [23]; total crude fat content was determined by the Soxhlet method [24]; and ash content was determined by the mineralization of samples at 550 ± 25 °C [25].

The fatty acid composition was determined according to O’Fallon et al. [26]. Fatty acid methyl esters (FAMEs) were analysed by gas chromatography (Shimadzu GC-2014, Shimadzu Corporation, Kyoto, Japan) equipped with a flame ionisation detector. Full details of sample preparation, derivatisation, and instrumental conditions are described by Stanišić et al. [27]. Individual FAMEs were identified by comparing retention times with those of a certified standard mixture (Supelco 37 Component FAME Mix, Sigma-Aldrich, Hamburg, Germany). Fatty acid concentrations were expressed as a percentage of total identified fatty acids. Saturated fatty acids (SFA), monounsaturated fatty acids (MUFA), and polyunsaturated fatty acids (PUFA) were calculated as the sum of their respective fatty acids.

### 2.3. Statistical Analysis

All statistical analyses were conducted using IBM SPSS Statistics v24 (IBM Corp., Armonk, NY, USA). The study employed a 2 × 2 factorial design with breed (Banat Naked Neck, BNN; Svrljig hen, SH) and slaughter age (12 and 14 weeks) as fixed factors. For each response variable (carcass traits, dissection yields, proximate composition, fatty acid traits, and physical properties), a two-way analysis of variance was performed using the General Linear Model (univariate) to test the main effects of breed and age and their interaction (breed × age). When a significant breed × age interaction was found, simple effects were examined using pairwise comparisons based on estimated marginal means with Sidak adjustment. Results are presented as mean ± standard deviation (Mean ± SD), and statistical significance was set at *p* < 0.05.

To explore relationships among multiple meat quality variables, principal component analysis (PCA) was performed separately for breast (white meat) and thigh-with-drumstick (dark meat) datasets. Variables were standardised (z-scores) prior to PCA to account for differences in measurement scales. PCA results were summarised by the proportion of variance explained by the principal components and visualised using biplots of the first two components (PC1 and PC2). Final PCA plots were formatted for publication in Microsoft Excel (Microsoft Corp., Redmond, WA, USA).

Associations among carcass part proportions, linear carcass measurements, and dissection components were evaluated using Spearman rank correlations after adjustment for confounding effects. For each variable, residuals were obtained from GLM (univariate) models including breed and age as fixed factors and slaughter live weight as a covariate. This residualisation procedure was applied to remove systematic variation attributable to breed, slaughter age and body size, so that the correlations would reflect net inter-trait associations independent of these confounding factors. Spearman correlations were then calculated among residuals to assess associations independent of breed, age, and body size. Thus, residual correlation analysis was used to identify trait covariation beyond the main experimental factors, rather than simple phenotypic correlations driven by differences in genotype, age or live weight. The resulting residual correlation matrix was visualised as a heatmap formatted in Microsoft Excel (Microsoft Corp., Redmond, WA, USA).

## 3. Results

In Table 1, slaughter age increased slaughter weight (SW) and conventionally dressed carcass (CC) weight, both of which were higher at 14 weeks than at 12 weeks (*p* < 0.001), while breed and the breed × age interaction had no effect on these traits (*p* > 0.05). Slaughter age also affected all carcass conformation measures, with shank length (SL), keel length (KL), breast depth (BD), thigh girth (TG) and breast angle (BA) all higher at 14 weeks (*p* ≤ 0.024). Breed effects were detected only for TG (*p* = 0.030) and BA (*p* = 0.007), and no breed × age interactions were observed for any conformation trait (*p* > 0.05). Similarly, all conformation indices (SW/SL, SW/KL, SW/BD and SW/TG) increased with slaughter age (all *p* < 0.001; Table 1). Neither breed nor the breed × age interaction affected these indices, although SW/BD showed a tendency towards a breed effect (*p* = 0.067).

The distribution of carcass parts and edible organs (% of SW) differed between breeds and slaughter ages for selected traits (Table 2). Head proportion was affected by slaughter age (*p* < 0.001) and showed a significant breed × age interaction (*p* = 0.007), while the main effect of breed was not significant (*p* = 0.214). Neck proportion differed between breeds (*p* < 0.001), with no effect of slaughter age and no breed × age interaction (*p* > 0.05). Among the main carcass cuts, back proportion was influenced by breed (*p* = 0.041), while metatarsus, pelvis, and wing proportions were not significantly affected by breed, age, or their interaction (*p* > 0.05). Breast proportion was significantly affected by breed (*p* = 0.001) and slaughter age (*p* = 0.045), and also showed a breed × age interaction (*p* = 0.017). The proportion of thigh with drumstick increased with slaughter age (*p* = 0.024). For edible organs, gizzard proportion differed between slaughter ages (*p* = 0.031), while heart and liver proportions were not significantly affected by breed, age, or their interaction (*p* > 0.05).

Breast dissection traits were influenced by breed and slaughter age for selected variables (Table 3). The proportion of breast meat as a percentage of the cut was higher in BNN than in SH (*p* = 0.032) and increased with slaughter age (*p* = 0.033), with no significant breed × age interaction (*p* = 0.628). When expressed relative to CC, breast meat yield differed by breed (*p* = 0.010) and slaughter age (*p* = 0.039), and a significant breed × age interaction was observed (*p* = 0.039), indicating that age-related changes in breast meat yield were not consistent between breeds. Breast bone proportion (% of cut) showed borderline effects of breed (*p* = 0.050) and age (*p* = 0.051), whereas bone yield relative to CC was not affected by breed, age, or their interaction (*p* > 0.05). Breast skin proportion and skin yield (both % of cut and % of CC) did not differ significantly by breed, slaughter age, or their interaction (*p* > 0.05).

For the thigh with drumstick part, slaughter age significantly affected meat yield, with higher values at 14 weeks both as a percentage of the cut (*p* = 0.003) and as a percentage of CC (*p* = 0.005), while breed and breed × age effects were not significant (*p* > 0.05). Bone proportion (% of cut) decreased with slaughter age (*p* = 0.030), whereas bone yield relative to CC was not significantly affected (*p* > 0.05), although the breed × age interaction showed a tendency (*p* = 0.086). Skin proportion and skin yield for thigh with drumstick were not significantly influenced by breed, slaughter age, or their interaction (*p* > 0.05).

**Figure 1:**
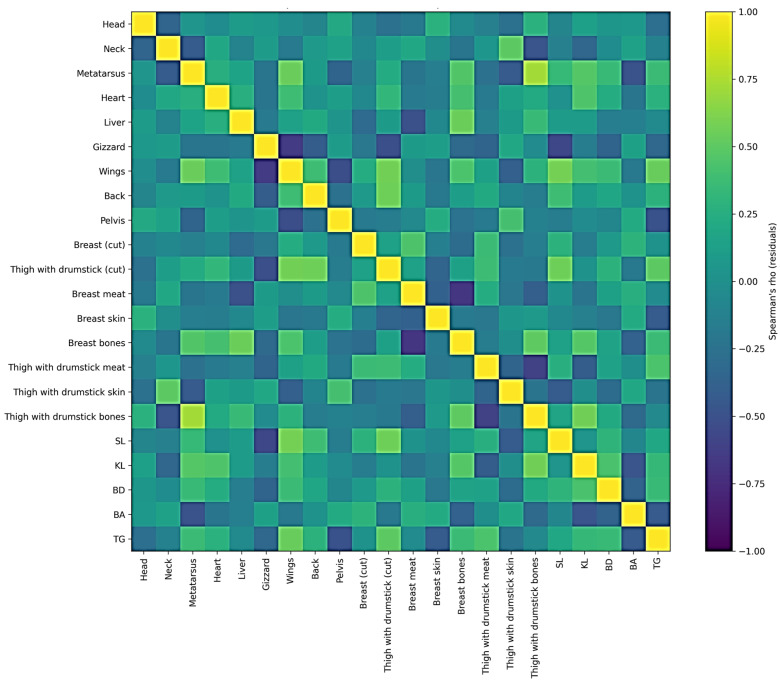
Residual Spearman correlation heatmap of carcass part yields, breast and thigh with drumstick dissection traits, and carcass conformation measures in Banat Naked Neck (BNN) and Svrljig hen (SH) chickens. Residuals were obtained from GLM models with breed and slaughter age as fixed factors and slaughter live weight as a covariate. Spearman’s rank correlations were then calculated among the residuals (colour scale: −1 to +1). The full correlation matrix is provided in Supplementary Table S1.

Figure 1 summarises the residual relationships among carcass part yields, dissection traits, and conformation measures after accounting for breed, age, and slaughter live weight. Several strong associations were evident (as shown in Supplementary Table S1). The strongest positive residual correlations (|ρ| ≥ 0.50) included Metatarsus–Thigh with drumstick bones (ρ = 0.719, *p* < 0.01), Wings–Shank length (SL) (ρ = 0.583, *p* < 0.01), Wings–Thigh with drumstick (cut) (ρ = 0.575, *p* < 0.01), Thigh with drumstick (cut)–Back (ρ = 0.555, *p* < 0.01), Liver–Breast bones (ρ = 0.552, *p* < 0.01), and Wings–Metatarsus (ρ = 0.541, *p* < 0.01). Additional notable positive links were observed between Leg bones–Keel length (KL) (ρ = 0.567, *p* < 0.01), Wings–Thigh girth (TG) (ρ = 0.536, *p* < 0.01), and Breast bones–Thigh with drumstick bones (ρ = 0.505, *p* < 0.01).

Conversely, several strong negative residual correlations (|ρ| ≥ 0.50) indicated clear trade-offs within cuts and between some carcass components. The most pronounced were Breast meat–Breast bones (ρ = −0.684, *p* < 0.01), Gizzard–Wings (ρ = −0.656, *p* < 0.01), Thigh with drumstick meat–Thigh with drumstick bones (ρ = −0.607, *p* < 0.01), and Gizzard–SL (ρ = −0.568, *p* < 0.01). In addition, Pelvis–Wings showed a strong negative association (ρ = −0.519, *p* < 0.01), and Gizzard–Thigh with drumstick (cut) was also negatively related (ρ = −0.514, *p* < 0.01). Finally, Liver–Breast meat showed a moderate-to-strong negative association (ρ = −0.502, *p* < 0.01).

As shown in Table 4, breast meat moisture content was affected by slaughter age (*p* = 0.003) and by the breed × age interaction (*p* = 0.023), whereas breed alone was not significant (*p* = 0.283). Intramuscular crude fat content differed between breeds (*p* = 0.040), while slaughter age and the interaction were not significant (*p* > 0.05). Crude protein content was influenced by slaughter age (*p* = 0.005) and by the breed × age interaction (*p* = 0.024), with no main effect of breed (*p* = 0.495). Ash content was not significantly affected by breed, slaughter age or their interaction (*p* > 0.05), although there was a tendency for an age effect (*p* = 0.059).

For the fatty acid profile of breast meat (Table 4), only selected fatty acids were affect-ed. C14:0 differed between breeds (*p* = 0.031) and showed a breed × age interaction (*p* = 0.047), while slaughter age alone was not significant (*p* = 0.394). The proportion of C18:3 n − 3 also differed between breeds (*p* = 0.025), with no age or interaction effect (*p* > 0.05). C22:6 n − 3 was influenced by slaughter age (*p* = 0.013) and by the breed × age interaction (*p* = 0.033), whereas breed was not significant (*p* = 0.447). C24:0 increased with slaughter age (*p* = 0.021), with no breed or interaction effect (*p* > 0.05) and C18:2 n − 6 showed a tendency towards a breed × age interaction (*p* = 0.066). No significant effects were found for total SFA, MUFA or PUFA (*p* > 0.05). Similarly, Σn − 6 and Σn − 3 were not significantly affected by breed or slaughter age, although Σn − 3 showed a tendency for interaction (*p* = 0.067). The Σn − 6/Σn − 3 ratio showed a significant breed × age interaction (*p* = 0.019), while the main effects of breed and slaughter age were not significant (*p* > 0.05).

As shown in Table 5, moisture content differed between breeds (*p* = 0.014) and slaughter ages (*p* = 0.017), with no breed × age interaction (*p* = 0.637). Intramuscular crude fat content was affected by breed (*p* < 0.001), whereas slaughter age and the breed × age interaction were not significant (*p* > 0.05). Crude protein and ash contents were not significantly influenced by breed, slaughter age, or their interaction (*p* > 0.05).

Several fatty acids were influenced by breed and/or slaughter age (Table 5). Slaughter age affected C14:0 (*p* = 0.008), C18:1 n − 7 (*p* < 0.001), and C24:1 n − 9 (*p* = 0.019), and showed a tendency for C24:0 (*p* < 0.001) and the n − 6/n − 3 ratio (*p* = 0.035). Breed effects were detected for C16:0 (*p* = 0.010) and C20:4 n − 6 (*p* = 0.009), as well as for total SFA (*p* = 0.022), while no significant breed effects were found for MUFA, PUFA, or Σn − 6 (*p* > 0.05). Significant breed × age interactions were observed for C16:1 (*p* = 0.009), C18:0 (*p* = 0.002), and C18:1 n − 7 (*p* = 0.018). In addition, Σn − 3 was influenced by slaughter age (*p* = 0.023) and showed a significant breed × age interaction (*p* < 0.001), whereas Σn − 6 and Σn − 6/Σn − 3 showed no interaction effects (*p* > 0.05). Overall, no significant effects of breed, slaughter age, or their interaction were found for total MUFA, PUFA, or Σn − 6 (*p* > 0.05).

**Figure 2:**
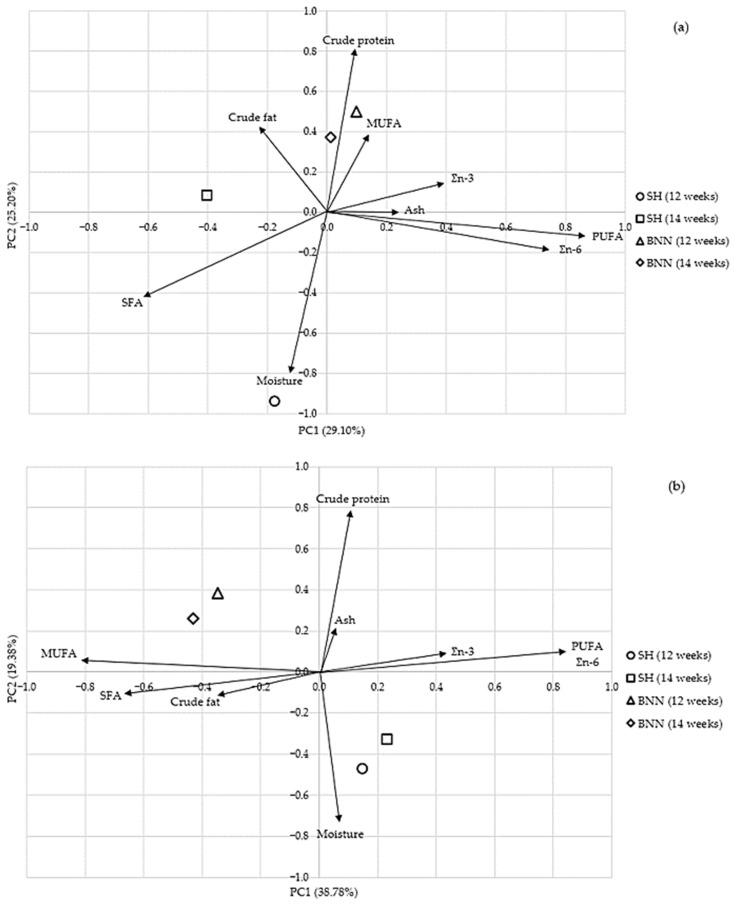
Principal component analysis (PCA) biplots based on proximate composition and major fatty acid groups in (**a**) breast meat and (**b**) thigh with drumstick meat of Banat Naked Neck (BNN) and Svrljig hen (SH) chickens slaughtered at 12 and 14 weeks of age. Vectors (arrows) represent variable loadings on PC1 and PC2, and points represent group centroids for each breed × age combination.

In Figure 2a, the PCA biplot for breast meat showed that the first two components explained 54.3% of the total variability (PC1 = 29.10%, PC2 = 25.20%). PC1 mainly reflected variation in fatty acid groups, with PUFA and Ʃn − 6 (and, to a lesser extent, Ʃn − 3 and ash) loading on the positive side and SFA on the negative side. PC2 was primarily associated with crude protein and MUFA versus moisture, which loaded strongly in the negative direction. Regarding group distribution, both BNN groups were positioned in the upper part of the plot, whereas SH at 12 weeks was clearly shifted downward. The SH 14-week group was also displaced towards the negative side of PC1 compared with the other groups.

In Figure 2b, the PCA for thigh-with-drumstick meat explained 58.2% of the total variability (PC1 = 38.78%, PC2 = 19.38%). PC1 again represented the main contrast among fatty acid groups, with PUFA and n − 6 showing strong positive loadings and MUFA, SFA, and crude fat loading on the negative side. PC2 separated samples mainly according to crude protein and ash (positive) versus moisture (negative). The score plot showed a clear breed separation along PC1, with both BNN groups on the negative side and both SH groups on the positive side. Along PC2, the SH groups clustered lower than the BNN groups, while the two age groups within each breed remained relatively close.

## 4. Discussion

The present dataset provides a clear comparison of two Serbian indigenous dual-purpose breeds under the same growing conditions, demonstrating that slaughter age was the main driver of carcass size and conformation (Table 1), while breed-related differences were more evident in carcass part distribution and tissue composition (Table 2 and Table 3) and in selected chemical and lipid traits, particularly in thigh with drumstick meat (Table 4 and Table 5).

Overall, these breeds demonstrate modest potential for meat production compared with fast-growing commercial broilers, and differences are apparent when compared with moderate- and slow-growing hybrids intended for free-range or traditional systems [28,29,30,31], as well as with other indigenous breeds [32]. The dual-purpose nature of BNN and SH partly explains their lower body weight relative to meat-type genotypes, along with the absence of long-term selection for body weight improvement (a trait with relatively high heritability). However, when comparing BNN performance with earlier reports [15], some improvement in body weight and carcass properties at similar slaughter ages can be noted. In addition to genetic improvement (selection and/or crossbreeding), adjustments in rearing, nutrition, and health care may further enhance production results [33]. This may partly explain deviations from earlier research [18], where higher body weights of BNN and SH males at 84 days were reported, likely supported by a more energy- and protein-dense diet (23 to 21.5% of crude proteins, 3050 to 3080 kcal ME/kg). Similar growth trajectories and final weights in BNN and SH suggest no major differences in adaptability to the applied growing conditions. Growth curves are often considered a more sensitive indicator of adaptability to free-range systems than a single pre-slaughter measurement [8]. In this study, both the weekly growth pattern and the final slaughter weight supported the conclusion of comparable adaptability. Regarding rearing duration, the results justify slaughter at an older age due to the consistent increase in slaughter weight and the improvement of several conformation traits with age (Table 1). Similarly, the effect of age on body weight has been confirmed in other indigenous genotypes (e.g., Oravka, a Slovakian indigenous breed) [34]. The two-factor ANOVA results for slaughter weight and conformation support these observations: slaughter age increased slaughter weight and all measured conformation traits (Table 1), whereas breed effects were limited to traits reflecting breast and leg development (breast angle and thigh girth), suggesting broadly comparable growth capacity but some differences in body shape between BNN and SH.

Carcass part distribution (Table 2) further highlighted breed- and age-related differences in anatomical allocation. In particular, breast proportion differed between breeds and was influenced by slaughter age, with a significant breed × age interaction, indicating that the age-related change in breast yield was not identical in BNN and SH. Thigh-with-drumstick proportion increased with slaughter age, supporting the practical benefit of extending rearing to 14 weeks for improving valuable cut yields in these genotypes. The most prevalent differences typically reported among local or indigenous breeds relate to breast and leg proportions, as well as head and neck components [35,36,37,38], which aligns with the pattern observed here. In addition, age effects on some non-carcass components (e.g., head and gizzard) indicate that body component “priority” continues to shift slightly between 12 and 14 weeks in these slow-growing birds (Table 2).

Dissection results (Table 3) provide further insight into carcass value. BNN generally exhibited a higher breast meat proportion (both as a percentage of cut and of CC), while slaughter age increased breast and thigh with drumstick meat yields, particularly when expressed relative to carcass weight. Notably, the significant breed × age interaction for breast meat yield (% of CC) suggests that SH benefitted more from the extended rearing period in terms of breast meat deposition, whereas BNN showed a more stable breast meat yield across ages. At the same time, bone proportion in the thigh with drumstick cut decreased with slaughter age, consistent with progressive muscle accretion outpacing bone growth during this phase. Using the percentage of leg bones as a criterion for evaluating skeletal robustness [11], the general similarity in leg bone yields supports comparable skeletal robustness between BNN and SH.

To complement the univariate comparisons in Table 1, Table 2 and Table 3, the residual Spearman correlation heatmap (Figure 1; Supplementary Table S1) summarises how carcass parts, dissection traits, and conformation measures covary after adjusting for breed, age, and slaughter live weight. By analysing correlations among residuals rather than raw values, the aim was to identify net relationships among traits that were not solely driven by differences in genotype, slaughter age, or body size. Several strong relationships (|ρ| ≥ 0.50) remained evident (as shown in Supplementary Table S1), suggesting biologically meaningful allocation patterns that persist beyond the main design factors. In particular, metatarsus showed a strong positive association with leg bones (ρ = 0.719) and was also positively related to wings (ρ = 0.541), indicating coordinated development of locomotor structures. Such associations likely reflect common underlying patterns of skeletal growth, in which birds with relatively more developed appendicular structures also tend to exhibit a greater relative proportion of other bone-related components. Wings were strongly negatively associated with gizzard (ρ = −0.656), while also correlating positively with thigh with drumstick part (ρ = 0.575) and shank length (ρ = 0.583), linking cut proportions with conformation traits. This may indicate that, after accounting for overall size and design factors, individuals still differ in how growth is allocated between locomotor or body conformation traits and visceral organ development. A pronounced inverse relationship between breast meat and breast bones (ρ = −0.684) reflected an expected within-cut trade-off, while other strong associations included back with thigh-with-drumstick (ρ = 0.555), gizzard with shank length (ρ = −0.568), and leg bones with keel length (ρ = 0.567). Similarly, the negative association between thigh-with-drumstick meat and bones suggests that a greater relative contribution of muscle within a cut is accompanied by a lower relative proportion of skeletal tissue, consistent with carcass partitioning trade-offs during growth. Overall, these correlations indicate that even under the same management conditions, individual birds differ in how tissue is distributed among structural components (shank/metatarsus), cut yields, and dissected tissues, and the residual-based approach shows that these patterns cannot be explained only by breed, slaughter age, or slaughter weight.

Studies comparing indigenous or local breeds with commercial broilers generally report higher dry matter and protein, and lower fat, in local breeds [5,32,36]. Analysis of amino acid composition also indicates that meat from local breeds may provide a favourable essential amino acid profile compared to broiler meat [39]. Such findings reflect genetic background and slower growth, but also higher activity, diet composition, and older slaughter age. In the present study, breed-specific differences were observed within the indigenous group itself, especially in dark meat. Breast and thigh meat of BNN showed higher crude fat content compared to SH (Table 4 and Table 5), and BNN thigh with drumstick meat also tended to show a fatty acid profile consistent with higher SFA and certain individual SFAs, while SH showed relatively higher arachidonic acid (C20:4 n − 6) (Table 5). Given that both breeds had the same formulated diet and access to pasture, these differences likely reflect genotype-dependent utilisation and metabolism of fatty acids. In particular, differences in the capacity to use natural α-linolenic acid sources (and downstream metabolism) may contribute to differences in Ʃn − 3 PUFA deposition [40,41]. Similar genotype-dependent differences in PUFA biosynthesis capacity have been reported among dual-purpose breeds and can persist even under comparable feeding systems [42]. Dalle Zotte et al. [28] also reported that leg meat of two Italian autochthonous breeds tends to show greater variability in composition and fatty acids than breast meat, which they linked to higher intramuscular fat levels in dark meat. The detailed lipid profiles support this breed- and cut-specific pattern. In breast meat (Table 4), differences between breeds were limited to selected fatty acids, while several traits displayed breed × age interactions (e.g., C14:0, C22:6 n − 3, and the Σn − 6/Σn − 3 ratio), suggesting that the effect of slaughter age on nutritional indices may depend on genotype. In thigh with drumstick meat (Table 5), breed effects were clearer for both proximate traits (notably moisture and crude fat) and for lipid fractions and specific fatty acids (e.g., C16:0, C20:4 n − 6, and SFA), while Σn − 3 increased with slaughter age and showed a strong interaction, consistent with the conclusion that leg meat is more sensitive for detecting genotype-related differences in fatty acid metabolism.

The multivariate PCA (Figure 2) integrates the proximate and fatty-acid data, clarifying patterns that are less apparent in the univariate tables. In breast meat (Figure 2a), the first two principal components captured variation mainly along a lipid-quality axis (PC1; 29.10% of variance), contrasting PUFA and Σn − 6/Σn − 3 with SFA and crude fat, and a compositional axis (PC2; 25.20%) contrasting moisture with crude protein. The position of group centroids suggests that differences among breast samples are relatively subtle and are driven more by the moisture–crude protein balance and age-related changes. This is consistent with the limited number of significant breed effects in breast meat (Table 4) and the presence of several breed × age interactions rather than consistent main effects. In contrast, the PCA for thigh with drumstick meat (Figure 2b) showed a clearer separation by breed along PC1 (38.78%), with SH centroids oriented toward PUFA/Σn − 6 loadings and BNN centroids clustering toward MUFA/SFA and crude fat loadings. This multivariate separation aligns with the univariate findings for dark meat (Table 5), where breed effects were more pronounced for proximate composition and several fatty acid traits, supporting the interpretation that genotype-related differences in lipid deposition and fatty acid profile are expressed more strongly in thigh with drumstick meat than in breast meat.

Overall, integrating the univariate results (Table 1, Table 2, Table 3, Table 4 and Table 5) with the multivariate outputs (Figure 1 and Figure 2) supports the conclusion that extending the slaughter age to 14 weeks consistently improves carcass conformation and meat yield-related traits, whereas breed-related differences are most evident in carcass part distribution, tissue partitioning within the breast and thigh-with-drumstick cuts, and the lipid profile of thigh meat. These findings are directly relevant for developing valorisation strategies to support the conservation of BNN and SH through market-oriented, non-conventional production systems. The sustainability of such systems is closely linked to consumer acceptance; generally, consumers maintain a positive attitude toward meat from non-conventional systems, perceiving it as healthier and possessing superior sensory attributes compared to meat from conventional production. The study findings suggest breed-related differences that may influence consumer preferences and specific quality requirements. However, further research is needed to provide a basis for the development of niche products and the enhancement of their market potential.

## 5. Conclusions

This study offers a comparative characterisation of carcass traits and meat composition in two Serbian indigenous chicken breeds: Banat Naked Neck (BNN) and Svrljig hen (SH), slaughtered at 12 and 14 weeks under identical rearing conditions. Extending the rearing period to 14 weeks consistently improved slaughter weight, conventionally dressed carcass weight and carcass conformation measures, supporting 14 weeks as a practical slaughter age when the aim is to increase carcass value in slow-growing systems. Breed-related differences were more evident in carcass part distribution and tissue composition of cuts, and were particularly pronounced in the compositional profile of thigh with drumstick meat, while differences in breast meat were generally smaller. The multivariate analyses (residual correlation heatmap and PCA) complemented the univariate analyses by illustrating coordinated relationships among carcass parts, conformation traits, and cut composition after accounting for the main design factors.

From an application perspective, the results suggest two clear directions for valorisation in non-conventional production systems: BNN appears more favourable when the priority is carcass conformation and breast-related yield, whereas SH shows distinctive traits in thigh with drumstick meat composition that may be attractive for differentiated market positioning. Future work should evaluate these breeds at older slaughter ages and under different non-conventional management conditions, include both sexes, and expand assessment to broader sensory and nutritional quality attributes to better support their conservation.

The broader implications of this study and results are their contribution to preservation of the gene pool of local chicken breeds and their importance for biodiversity.

## Figures and Tables

**Table 1:** Slaughter weight, conventionally dressed carcass weight and carcass conformation traits (measures and indices) of Banat Naked Neck (BNN) and Svrljig hen (SH) chickens slaughtered at 12 and 14 weeks of age.

	BNN		SH		*p*-Value		
	12	14	12	14	Breed	Age	B × A
SW, g	1226.10 ± 79.13	1457.10 ± 157.93	1092.90 ± 224.70	1461.10 ± 206.62	0.254	<0.001	0.227
CC, g	1000.09 ± 58.28	1206.51 ± 139.47	860.36 ± 194.89	1171.12 ± 174.33	0.075	<0.001	0.282
Conformation measures							
SL, mm	84.00 ± 4.85	91.00 ± 5.77	78.20 ± 7.93	90.00 ± 5.62	0.089	<0.001	0.225
KL, mm	104.40 ± 4.03	108.20 ± 5.61	99.10 ± 7.32	107.50 ± 11.25	0.217	0.015	0.342
BD, mm	96.40 ± 5.40	99.60 ± 8.03	92.80 ± 7.80	103.10 ± 7.26	0.982	0.005	0.128
TG, mm	112.50 ± 4.03	119.30 ± 3.40	106.10 ± 8.49	116.50 ± 7.96	0.030	<0.001	0.379
BA, degree	96.10 ± 3.96	101.40 ± 6.33	93.20 ± 4.71	94.90 ± 5.63	0.007	0.024	0.284
Conformation indices							
SW/SL, g/mm	14.62 ± 0.98	16.00 ± 1.25	13.84 ± 1.67	16.21 ± 1.91	0.547	<0.001	0.304
SW/KL, g/mm	11.74 ± 0.58	13.46 ± 0.96	11.00 ± 1.93	13.57 ± 1.05	0.420	<0.001	0.280
SW/BD, g/mm	12.77 ± 0.86	14.65 ± 1.31	11.69 ± 1.70	14.14 ± 1.30	0.067	<0.001	0.511
SW/TG, g/mm	10.90 ± 0.66	12.21 ± 1.23	10.23 ± 1.58	12.50 ± 1.15	0.616	<0.001	0.211

SW, slaughter weight (g); CC, conventionally dressed carcass (g); SL, shank length (mm); KL, keel length (mm); BD, breast depth (mm); TG, thigh girth (mm); BA, breast angle (°). Conformation indices are expressed as SW/measure (g/mm).

**Table 2:** Proportions of carcass parts and edible organs (% of slaughter weight) in Banat Naked Neck (BNN) and Svrljig hen (SH) chickens slaughtered at 12 and 14 weeks of age.

	BNN		SH		*p*-Value		
	12	14	12	14	Breed	Age	B × A
Head	3.76 ± 0.54	3.55 ± 0.29	4.71 ± 1.30	3.18 ± 0.20	0.214	<0.001	0.007
Neck	4.28 ± 0.30	4.11 ± 0.31	4.57 ± 0.48	4.77 ± 0.40	<0.001	0.871	0.124
Metatarsus	4.50 ± 0.40	4.63 ± 0.41	4.64 ± 0.47	4.28 ± 0.56	0.494	0.449	0.105
Back	8.34 ± 0.88	8.39 ± 0.46	7.85 ± 0.56	7.96 ± 0.76	0.041	0.707	0.911
Pelvis	8.34 ± 0.81	8.58 ± 0.34	7.85 ± 0.74	8.37 ± 0.60	0.100	0.073	0.499
Wings	9.23 ± 0.60	9.27 ± 0.39	9.09 ± 0.58	8.88 ± 0.37	0.100	0.566	0.428
Breast	15.60 ± 1.22	15.44 ± 0.91	13.31 ± 1.61	15.04 ± 0.89	0.001	0.045	0.017
Thigh with drumstick	21.63 ± 0.70	22.59 ± 1.00	21.12 ± 1.80	21.94 ± 1.02	0.136	0.024	0.856
Heart	0.51 ± 0.12	0.49 ± 0.05	0.51 ± 0.05	0.44 ± 0.07	0.394	0.094	0.272
Liver	2.04 ± 0.31	1.87 ± 0.28	2.05 ± 0.35	1.90 ± 0.18	0.820	0.096	0.883
Gizzard	2.92 ± 0.26	2.81 ± 0.76	3.20 ± 0.49	2.54 ± 0.49	0.975	0.031	0.110

**Table 3:** Dissection yields of breast and thigh with drumstick (meat, bone, and skin) expressed as percentage of the cut and as percentage of conventionally dressed carcass (CC) in Banat Naked Neck (BNN) and Svrljig hen (SH) chickens slaughtered at 12 and 14 weeks of age.

	BNN		SH		*p*-Value		
	12	14	12	14	Breed	Age	B × A
Breast							
Meat (% of cut)	65.00 ± 2.62	66.56 ± 2.14	62.54 ± 4.06	64.98 ± 2.16	0.032	0.033	0.628
Meat (% of CC)	12.42 ± 1.02	12.42 ± 0.84	10.66 ± 1.62	12.22 ± 0.95	0.010	0.039	0.039
Bones (% of cut)	23.75 ± 2.51	23.04 ± 2.06	26.50 ± 3.54	23.75 ± 2.49	0.050	0.051	0.241
Bones (% of CC)	4.53 ± 0.48	4.30 ± 0.44	4.46 ± 0.45	4.38 ± 0.34	0.964	0.262	0.593
Skin (% of cut)	7.38 ± 0.92	7.43 ± 0.67	7.44 ± 1.26	7.50 ± 1.04	0.831	0.853	0.986
Skin (% of CC)	1.41 ± 0.18	1.39 ± 0.14	1.26 ± 0.22	1.41 ± 0.23	0.333	0.293	0.163
Thigh with drumstick							
Meat (% of cut)	62.01 ± 1.38	63.66 ± 1.99	61.20 ± 3.11	63.88 ± 1.55	0.659	0.003	0.444
Meat (% of CC)	16.44 ± 0.70	17.38 ± 0.97	16.50 ± 1.46	17.51 ± 0.89	0.785	0.005	0.918
Bones (% of cut)	24.28 ± 1.76	23.81 ± 1.73	25.59 ± 2.78	23.17 ± 1.57	0.600	0.030	0.135
Bones (% of CC)	6.43 ± 0.44	6.50 ± 0.59	6.87 ± 0.61	6.35 ± 0.48	0.406	0.193	0.086
Skin (% of cut)	9.81 ± 1.25	9.08 ± 1.69	8.57 ± 1.59	9.31 ± 1.43	0.290	0.987	0.132
Skin (% of CC)	2.60 ± 0.33	2.48 ± 0.45	2.31 ± 0.49	2.54 ± 0.35	0.402	0.678	0.181

**Table 4:** Proximate composition and fatty acid profile of breast meat in Banat Naked Neck (BNN) and Svrljig hen (SH) chickens slaughtered at 12 and 14 weeks of age.

	BNN		SH		*p*-Value		
	12	14	12	14	Breed	Age	B × A
Proximate composition (%)							
Moisture	74.41 ± 0.60	74.22 ± 0.85	75.15 ± 0.59	73.96 ± 0.71	0.283	0.003	0.023
Crude fat	0.50 ± 0.12	0.54 ± 0.13	0.41 ± 0.11	0.46 ± 0.13	0.040	0.278	0.837
Crude protein	24.10 ± 0.61	24.24 ± 0.76	23.45 ± 0.53	24.59 ± 0.76	0.495	0.005	0.024
Ash	0.78 ± 0.41	1.00 ± 0.05	0.88 ± 0.29	0.98 ± 0.05	0.625	0.059	0.464
Fatty acid composition (% of total FAs)							
C14:0	0.53 ± 0.21	0.46 ± 0.15	0.55 ± 0.20	0.74 ± 0.25	0.031	0.394	0.047
C16:0	30.10 ± 0.41	29.53 ± 4.20	29.57 ± 3.36	29.82 ± 4.00	0.920	0.899	0.732
C16:1	1.84 ± 0.50	1.66 ± 0.69	1.41 ± 0.65	1.78 ± 0.90	0.478	0.681	0.221
C18:0	9.63 ± 1.03	9.35 ± 2.94	10.63 ± 1.49	8.97 ± 1.55	0.604	0.114	0.253
C18:1 n − 9	21.87 ± 2.04	22.40 ± 2.47	21.48 ± 2.44	20.43 ± 4.39	0.219	0.789	0.408
C18:1 n − 7	1.90 ± 0.73	1.82 ± 0.58	1.82 ± 0.58	1.43 ± 1.28	0.384	0.376	0.570
C18:2 n − 6	23.35 ± 1.46	24.93 ± 2.66	23.74 ± 2.80	21.61 ± 4.62	0.143	0.784	0.066
C18:3 n − 3	0.91 ± 0.27	1.10 ± 0.26	1.15 ± 0.41	1.42 ± 0.51	0.025	0.063	0.721
C20:4 n − 6	7.84 ± 1.36	7.72 ± 1.22	7.32 ± 2.37	7.94 ± 2.77	0.814	0.701	0.567
C22:6 n − 3	1.04 ± 0.45	0.53 ± 0.28	0.72 ± 0.28	0.68 ± 0.28	0.447	0.013	0.033
C24:0	0.64 ± 0.35	1.94 ± 1.42	0.60 ± 0.41	1.35 ± 1.20	0.466	0.021	0.524
C24:1 n − 9	1.18 ± 0.46	1.34 ± 0.71	0.88 ± 0.58	1.39 ± 0.97	0.584	0.137	0.437
SFA	40.89 ± 3.63	41.28 ± 4.48	41.34 ± 2.64	40.88 ± 3.23	0.982	0.976	0.708
MUFA	26.79 ± 2.80	27.20 ± 23.12	25.58 ± 2.64	25.03 ± 3.78	0.094	0.950	0.622
PUFA	33.13 ± 2.23	34.28 ± 3.07	32.92 ± 2.93	31.65 ± 4.69	0.189	0.951	0.261
Ʃn − 6	31.19 ± 2.00	32.65 ± 3.16	31.05 ± 2.84	29.50 ± 5.43	0.114	0.983	0.146
Ʃn − 3	1.95 ± 0.52	1.63 ± 0.22	1.87 ± 0.53	2.10 ± 0.51	0.180	0.762	0.067
Ʃn − 6/Ʃn − 3	17.02 ± 4.26	20.46 ± 3.68	18.28 ± 7.18	14.45 ± 2.26	0.119	0.898	0.019

**Table 5:** Proximate composition and fatty acid profile of thigh with drumstick meat in Banat Naked Neck (BNN) and Svrljig hen (SH) chickens slaughtered at 12 and 14 weeks of age.

	BNN		SH		*p*-Value		
	12	14	12	14	Breed	Age	B × A
Proximate composition (%)							
Moisture	75.58 ± 0.78	75.09 ± 0.57	76.32 ± 0.93	75.60 ± 0.74	0.014	0.017	0.637
Crude fat	2.60 ± 0.28	2.55 ± 0.59	1.66 ± 0.69	2.06 ± 0.60	<0.001	0.330	0.212
Crude protein	20.86 ± 0.83	21.27 ± 0.87	21.06 ± 1.27	21.35 ± 0.87	0.664	0.259	0.849
Ash	0.95 ± 0.04	0.98 ± 0.04	0.96 ± 0.06	0.98 ± 0.09	0.760	0.165	0.922
Fatty acid composition (% of total FAs)							
C14:0	0.52 ± 0.15	0.81 ± 0.36	0.61 ± 0.20	0.79 ± 0.28	0.674	0.008	0.532
C16:0	26.72 ± 2.73	26.02 ± 3.08	23.71 ± 2.08	24.43 ± 2.72	0.010	0.995	0.406
C16:1	4.18 ± 0.99	3.11 ± 1.29	2.58 ± 0.92	3.62 ± 1.48	0.156	0.964	0.009
C18:0	7.50 ± 1.27	8.77 ± 1.77	9.95 ± 1.88	7.33 ± 2.34	0.396	0.256	0.002
C18:1 n − 9	25.88 ± 1.21	26.48 ± 2.08	25.24 ± 2.53	25.75 ± 2.26	0.302	0.406	0.946
C18:1 n − 7	2.65 ± 0.66	1.21 ± 0.82	2.08 ± 0.44	1.73 ± 0.79	0.905	<0.001	0.018
C18:2 n − 6	28.48 ± 2.48	29.21 ± 3.82	28.93 ± 4.84	28.85 ± 4.50	0.975	0.801	0.752
C18:3 n − 3	1.43 ± 0.17	1.53 ± 0.45	1.48 ± 0.36	1.75 ± 0.47	0.280	0.139	0.465
C20:4 n − 6	2.47 ± 0.95	2.18 ± 0.69	3.65 ± 1.89	3.68 ± 2.13	0.009	0.792	0.741
C22:6 n − 3	0.23 ± 0.13	0.45 ± 0.23	0.40 ± 0.33	0.44 ± 0.15	0.251	0.087	0.219
C24:0	0.72 ± 0.40	1.08 ± 0.74	0.35 ± 0.20	1.26 ± 0.53	0.544	<0.001	0.092
C24:1 n − 9	0.34 ± 0.12	0.57 ± 0.24	0.42 ± 0.27	0.55 ± 0.27	0.665	0.019	0.508
SFA	35.46 ± 2.33	36.67 ± 2.82	34.62 ± 2.32	33.80 ± 2.34	0.022	0.806	0.203
MUFA	33.04 ± 1.86	31.37 ± 3.01	30.32 ± 3.52	31.63 ± 3.81	0.224	0.859	0.141
PUFA	32.62 ± 2.71	33.36 ± 4.43	34.45 ± 4.64	34.71 ± 5.19	0.254	0.718	0.861
Ʃn − 6	30.95 ± 2.62	31.39 ± 4.18	32.58 ± 4.55	32.53 ± 4.88	0.300	0.884	0.855
Ʃn − 3	1.66 ± 0.14	1.97 ± 0.54	1.88 ± 0.36	2.18 ± 0.48	0.105	0.023	<0.001
Ʃn − 6/Ʃn − 3	18.64 ± 1.29	16.83 ± 4.20	17.83 ± 3.62	15.27 ± 2.66	0.241	0.035	0.713

## Data Availability

The original contributions presented in this study are included in the article/Supplementary Material. Further inquiries can be directed to the corresponding author.

## Supplementary Materials

The following supporting information can be downloaded at: https://www.mdpi.com/article/10.3390/foods15091546/s1, Supplementary Table S1: Residual Spearman correlation matrix for carcass part proportions, dissection traits, and carcass conformation measures after adjustment for breed, slaughter age, and slaughter live weight (n = 40). Residuals were derived from GLM models; correlations represent associations independent of genotype, age, and body size. SL, shank length; KL, keel length; BD, breast depth; BA, breast angle; TG, thigh girth. *p* < 0.05; *p* < 0.01 (two-tailed).
